# Association of insomnia and daytime sleepiness with low back pain: A bidirectional mendelian randomization analysis

**DOI:** 10.3389/fgene.2022.938334

**Published:** 2022-10-04

**Authors:** Peng Shu, Lixian Ji, Zichuan Ping, Zhibo Sun, Wei Liu

**Affiliations:** ^1^ Department of Orthopedic Surgery, The Fourth Affiliated Hospital, Zhejiang University School of Medicine, Yiwu, China; ^2^ Department of Rheumatology, The Fourth Affiliated Hospital, Zhejiang University School of Medicine, Yiwu, China; ^3^ Department of Orthopedics, Renmin Hospital, Wuhan University, Wuhan, China

**Keywords:** insomnia, daytime sleepiness, low back pain, single nucleotide polymorphism, mendelian, randomization, causal association

## Abstract

**Purpose:** Observational research has indicated the presence of a causal relationship between sleep disturbances and low back pain (LBP). However, the link may have been biased by confounding factors. The purpose of this study was to examine the potential causal association of insomnia and daytime sleepiness with LBP by using mendelian randomization (MR).

**Methods:** Genome-wide association study (GWAS) summary statistics of insomnia were obtained from a large-scale GWAS meta-analysis (*n* = 1,331,010; individuals from UK Biobank and 23andMe) or UK Biobank alone (*n* = 453,379). The summary statistics of daytime sleepiness were from UK Biobank (*n* = 452,071) and LBP were provided by the FinnGen Release 6 (210,645 individuals with 16,356 LBP cases and 194,289 controls) or UK Biobank (5,423 cases versus 355,771 controls). Linkage disequilibrium score (LDSC) regression and bidirectional MR analysis was employed to estimate genetic correlation and causal relationship. In the MR analysis, the inverse variance weighted method (IVW) was utilized as the main analysis procedure, while MR-Egger, Weighted median and Robust adjusted profile score (RAPS) were utilized for supplementary analyses.

**Results:** LDSC analysis showed that LBP were significantly genetically correlated with insomnia (rg = 0.57, *p* = 2.26e-25) and daytime sleepiness (rg = 0.18, *p* = 0.001). The MR analysis revealed that genetically predicted insomnia was significantly associated with an increased risk of LBP (OR = 1.250, 95% CI: 1.186–1.318; *p* = 1.69e-16). However, the reverse causality was not confirmed. No evidence was identified supporting causality of daytime sleepiness and LBP.

**Conclusion:** This study demonstrates a putative causal link of insomnia on LBP and a null causal effect of LBP on insomnia. Furthermore, a causal link between daytime sleepiness and LBP were not reported. This finding may stimulate new strategies for patient management in clinical practice, benefiting public health.

## Introduction

Low back pain (LBP) is one of the most common patient complaints in orthopedic outpatient departments and imposes a high burden on individuals and society. According to The Global Burden of Disease study 2013, chronic LBP is the most frequent cause of disability worldwide (Global Burden of Disease Study 2013 Collaborators, 2015). The prevalence of LBP has been estimated between 60% and 80% (Hagen et al., 1997). Chronic LBP is associated with many risk factors and diseases, such as overweight and obesity, heavy physical effort and depression (Nieminen et al., 2021). Nonetheless, its etiology is multifarious, and in 90% of back pain patients, the underlying reason cannot be identified. Therefore, better identification of LBP risk factors could help advance public health and reduce the cost of treatment.

Insomnia, a frequently occurring sleep disorder characterized by trouble falling and/or staying asleep, is commonly reported by LBP patients, with a prevalence of at least 50% (Alsaadi et al., 2013). A cohort study by Agmon and coworkers documented that after adjusting for several potential confounding variables, such as socioeconomic status, self-rated health, lifestyle behavior, and anthropometrics, healthy employed adults are almost 1.5 times more likely to suffer from LBP after developing insomnia (Agmon and Armon, 2014). Daytime sleepiness, defined by having trouble staying awake during the day, is also associated with the increasing frequency of back pain (Gustafsson et al., 2018). Although many observational studies have revealed a bidirectional relationship between sleep disturbances and LBP, the causal association are unclear due to the confounders (Kelly et al., 2011; Goforth et al., 2014; Uhlig et al., 2018; Ho et al., 2019; Bilterys et al., 2021; Oliveira et al., 2022). A recent review focused on the association between sleep and spinal pain (including LBP) showed weak to moderate evidence of causality (Van Looveren et al., 2021). Thus, further research with more advanced methodology is necessary to establish a cause-and-effect link exists between LBP and sleep disturbances.

Traditional observational studies are inevitably biased since they are subject to many confounding factors. On the other hand, large randomized trials, which can provide the best evidence for causation, are expensive and time-consuming. Mendelian randomization (MR) provides an ingenious method for inferring the causal nature of the exposure-outcome relationship by using instrumental variables (IVs), such as single nucleotide polymorphisms (SNPs) (Gao et al., 2021; Zhou et al., 2022). Due to the random segregation of alleles at conception, genetic variants are fixed and not affected by environmental risk factors, which minimizes residual confounding and reverse causality. Therefore, MR is also considered as a “natural” randomized controlled trial.

Previous MR studies have successfully confirmed that sleep traits contributed to the risk of several diseases including psychiatric disorder (Gao et al., 2019), cardiovascular diseases (Yuan et al., 2021), amyotrophic lateral sclerosis (Zhang et al., 2021) and even osteoarthritis (Ni et al., 2022). Thus, the present study aimed to corroborate the putative causal link of insomnia and daytime sleepiness with LBP using bidirectional MR.

## Materials and methods

### Study design

**FIGURE 1:**
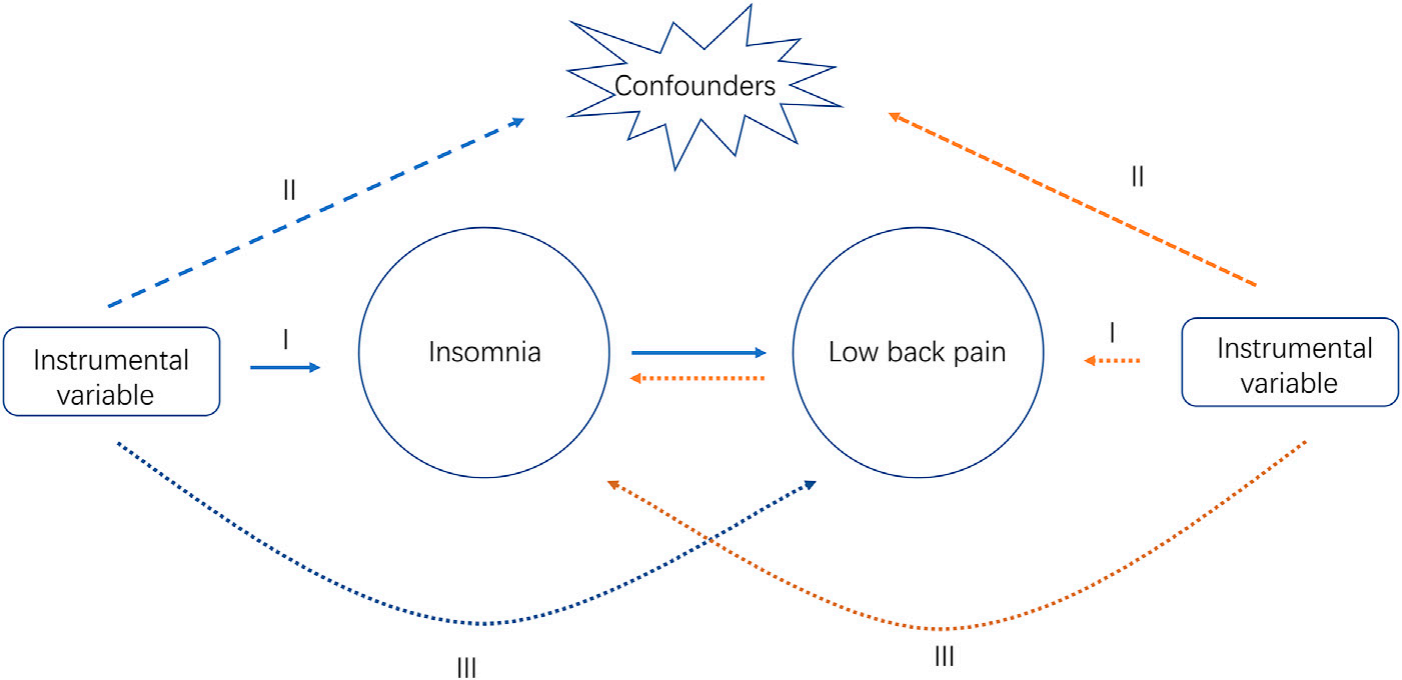
Study design overview. Ⅰ, Ⅱ, Ⅲ, represent the three key assumptions of MR design. The blue arrows represent the forward study whereas the yellow arrows represent the reverse study.

The overall design of the bidirectional MR study is shown in Figure 1. We selected SNPs as the genetic instrument and performed the MR analysis by the three key assumptions of MR design: 1) Relevance, i.e., genetic IVs are strongly associated with the exposure (Assumptions Ⅰ); 2) Independence, i.e., IVs are independent of any confounders (Assumptions Ⅱ); and 3) Exclusiveness, i.e., IVs do not affect the outcome directly, only possibly indirectly *via* the exposure (Assumptions Ⅲ) (Burgess et al., 2013). Robust MR methods with different model assumptions were utilized, such as inverse variance weighted (IVW), MR-Egger, Weighted median and Robust adjusted profile score (RAPS). Subsequently, we performed a series of analyses to identify potential pleiotropy and heterogeneity. Since all analyses in our study were based on publicly available GWAS datasets, ethics approval was not required.

### Genome-wide association study data sources

All IVs were retrieved from publicly available GWAS summary data. Our studies were restricted to subjects of European ancestry to minimize confounding caused by population stratification. The insomnia GWAS was reported by Jansen and collaborators and represents a large-scale (*N* = 1,331,010 individuals from UK Biobank and 23andMe) GWAS meta-analysis of insomnia from individuals of European ancestry (Jansen et al., 2019). The sample size of this data was 109,402 cases and 277,131 controls in the UK Biobank and 288,557 cases and 655,920 controls in the 23andMe database. Summary genetic statistic data for self-reported daytime sleepiness were available from the UK Biobank (452,071 individuals) (Wang et al., 2019). The summary statistics from GWAS for LBP were retrieved from the FinnGen Release 6 (R6), which includes 16,356 cases and 194,289 non-cases of European ancestry.

Importantly, the (Lane et al., 2019) summary statistics for insomnia were used for outcome data in our reverse research, because of it is higher powered than the Jansen data without 23andMe data. In the validation analysis, genetic association data for LBP from UK Biobank data were also used on the web https://gwas.mrcieu.ac.uk/datasets/ukb-d-M13_LOWBACKPAIN. Finally, the exposure and outcome data were rearranged according to effect alleles to obtain harmonized datasets for the next step in the analysis. The characteristics of participants and additional information about the GWAS datasets are listed in Supplementary Table S1.

### Genetic correlation analysis

Genetic correlations (rg) of insomnia, daytime sleepiness with LBP were estimated using Linkage disequilibrium score regression (LDSC) software package (https://github.com/bulik/ldsc) (Bulik-Sullivan et al., 2015).

### Mendelian randomization analysis

First, we evaluated the potential issue of weak IVs in MR analysis by performing the F-statistics. A mean F*-*statistics markedly greater than 10 usually indicates a small instrument bias (Bowden et al., 2016). Next, the conventional fixed-effect IVW method, which has the highest precision and assumes any horizontal pleiotropy is balanced, was implemented for the main analyses. The methods which included MR-Egger, Weighted median and RAPS were also employed for MR sensitivity analyses. Weighted median method can provide consistent estimates when at least half of the IVs are valid (Burgess et al., 2019). When there is pleiotropy in IVs, the MR-Egger method can obtain an effective estimation and the intercept in the MR-Egger regression can also detect pleiotropy (Bowden et al., 2015). RAPS method is robust to weak genetic IVs and can produce approximately unbiased results (Zhao et al., 2020). Cochran’s Q-test was used to detect potential heterogeneity. We also performed the MR-PRESSO analysis (SignifThreshold = 1) to detect outliers. If the MR-PRESSO outlier test calculated a *p* value less than the threshold, the outlier SNPs were removed. We performed the leave-one-out analysis to further identify potentially influential SNPs. Scatter plot and forest plot of the MR analyses were used to visualize the results.

All data analyses were conducted using the “Two-Sample MR” and “MR-PRESSO” packages in R version 4.0.2. Two-tailed *p* values < 0.05 were considered statistically significant putative causal link. A power calculation was performed using the online website (https://sb452.shinyapps.io/power/) developed by Burgess and coworkers (Burgess, 2014).

## Results

### Linkage disequilibrium score regression

Using LDSC regression, we found that LBP were significantly genetically correlated with insomnia and daytime sleepiness, with the highest correlation seen for insomnia (rg = 0.57, *p* = 2.26e-25) and the lowest for changes in daytime sleepiness (rg = 0.18, *p* = 0.001) (Table 1).

**TABLE 1:** Genetic correlation estimates from LDSC regression.

Phenotype 1	Phenotype 2	Rg (SE)	Pval
Insomnia	LBP	0.57 (0.05)	2.26e-25
Daytime sleepiness	LBP	0.18 (0.05)	0.001

Rg, genetic correlation; Pval, *p*-value for rg; SE, the standard error of Rg.

### IVs selection for mendelian randomization analysis

Firstly, we adopted the Jansen et al. insomnia dataset, in which 248 independent SNPs were identified as IVs of insomnia from combined results of the UK Biobank and 23andMe. Then, to satisfy the assumptions Ⅱ, a total of 158 independent SNPs was screened out after the LD test based on Europeans (*r* ^2^ < 0.001). Unless otherwise specified, significance threshold *p* of < 5 × 10^−8^ and the absence of linkage disequilibrium (LD) (cut-off *r* ^2^ < 0.001, clump window > 10,000 kb) were selected in our MR analysis. Under the same conditions, 38 SNPs as IVs of daytime sleepiness were obtained. In the reverse direction study, SNPs were identified to associate with LBP at a broadened threshold (*p* < 5 × 10^−6^) (Wu et al., 2021; Zhou et al., 2022). We extracted 26 SNPs from the FinnGen study and 20 SNPs from UK Biobank, respectively. The information of these SNPs is detailed in Supplementary Table S2.

### The causal effect of insomnia on low back pain

Initially, 4 SNPs of 158 lead SNPs were not found in the summary statistics for LBP (rs1064939, rs11001276, rs2286729, and rs73671843). Subsequently, nine SNPs (rs12991815, rs1731951, rs2030672, rs2221119, rs4858708, rs6119267, rs7044885, rs8180817, and rs830716) were removed for being palindromic with intermediate allele frequencies. For the remaining 145 SNPs, MR-PRESSO test detected seven potential outliers (rs1038093, rs12251016, rs12666306, rs429358, rs66674044, rs671985, and rs77641763). Finally, 138 SNPs as IVs of insomnia were selected to perform the MR analysis.

**FIGURE 2:**
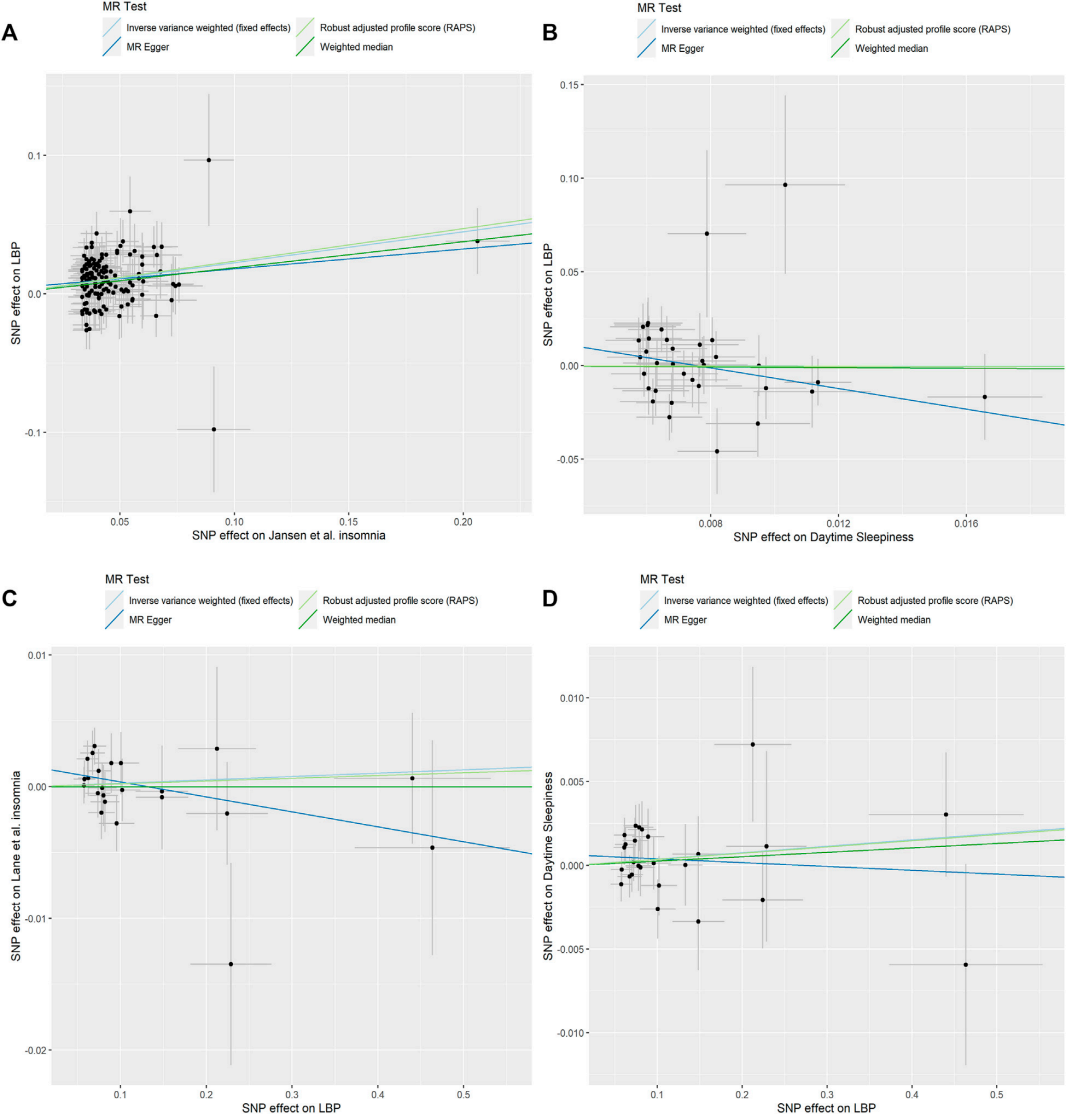
Scatter plots of causality. **(A)** Jansen et al. Insomnia on LBP. **(B)** Daytime Sleepiness on LBP. **(C)** LBP on Lane et al. Insomnia. **(D)** LBP on Daytime Sleepiness.

Our MR analyses showed that genetically predicted insomnia was causally associated with an increased risk of LBP. By using fixed-effect IVW method, the OR of LBP per genetically predicted insomnia was 1.25 [95% confidence interval (CI), 1.186–1.318; *p* = 1.69e-16] (Table 2). This statistically significant trend was further proven by Weighted median (OR = 1.21, CI: 1.105–1.318; *p* = 2.98e-05), RAPS (OR = 1.27, CI: 1.192–1.343; *p* = 1.08e-14) except for MR-Egger method (OR = 1.15, CI: 0.937–1.421; *p* = 0.179) (Table 2). The MR-Egger regression analysis did not indicate the presence of pleiotropy (intercept *p* = 0.435) and Cochran’s Q test did not detect significant heterogeneity in both MR-Egger and IVW methods (*p* > 0.05) (Supplementary Table S3). The causal effect of insomnia on LBP is illustrated by scatter plot (Figure 2A). Based on the effect size analysis, each insomnia SNP appeared to have a robust effect on LBP (Supplementary Figure S1). Furthermore, the leave-one-out analysis indicated that the significant result was not driven by any single SNP (Supplementary Figure S2). The mean F-statistics for IVs of insomnia is 43.73 in our study, which had ∼100% power to detect a 25% increase in overall LBP risk (OR = 1.25). Altogether, these results indicate that our data are robust and without obvious bias.

**TABLE 2:** Causal correlation estimates from MR analysis.

Method	nSNP	Beta	SE	Pval	OR	OR_lci95	OR_uci95
Insomnia on LBP							
MR Egger	138	0.143	0.106	0.179	1.154	0.937	1.421
Weighted median	138	0.188	0.043	1.47e-05	1.207	1.108	1.313
IVW	138	0.223	0.027	1.69e-16	1.250	1.186	1.318
RAPS	138	0.235	0.030	1.08e-14	1.266	1.192	1.343
Daytime sleepiness on LBP							
MR Egger	36	−2.593	1.637	0.123	0.075	0.003	1.851
Weighted median	36	−0.315	0.468	0.500	0.729	0.292	1.824
IVW	36	−0.151	0.318	0.636	0.860	0.461	1.606
RAPS	36	−0.147	0.399	0.713	0.863	0.395	1.887
LBP on insomnia							
MR Egger	23	−0.011	0.009	0.235	0.989	0.971	1.007
Weighted median	23	−3.09e-05	0.007	0.996	1.000	0.986	1.014
IVW	23	0.003	0.005	0.573	1.003	0.994	1.012
RAPS	23	0.002	0.005	0.663	1.002	0.993	1.012
LBP on daytime sleepiness							
MR Egger	26	−0.002	0.007	0.739	0.998	0.985	1.011
Weighted median	26	0.003	0.005	0.592	1.003	0.993	1.012
IVW	26	0.004	0.003	0.235	1.004	0.998	1.010
RAPS	26	0.004	0.004	0.303	1.004	0.997	1.011

nSNP, number of single-nucleotide polymorphism; Beta, the regression coeficient; SE, the standard error of the effect size; IVW, inverse-variance weighted; RAPS, Robust adjusted profile score.

### The causal effect of daytime sleepiness on low back pain

For 38 SNPs, one SNP (rs11078398) was not found in the summary statistics for LBP and one SNP (rs2048522) was removed for being palindromic. In the MR-PRESSO test, no outliers were detected. Finally, 36 SNPs as IVs of daytime sleepiness remained in this part of the analysis. No genetic effect of daytime sleepiness on the risk of LBP was found by IVW method (OR = 0.86, CI: 0.461–1.606; *p* = 0.636), and the other methods were concordant with this result (Figure 2B; Table 2). MR-Egger regression analysis did not indicate that 36 IVs had horizontal pleiotropy (intercept *p* = 0.135). Cochran’s Q test did not report any heterogeneity (*p* > 0.05) (Supplementary Table S3). Supplementary Figures S3, S4 shows the forest plot and the plot of leave-one-out analysis.

### The causal effect of low back pain on insomnia

All 26 SNPs were available for insomnia datasets and three SNPs (rs34960666, rs35989721, and rs4024198) were found as a potential outlier by MR-PRESSO analysis. All MR methods did not support significant causal effect of LBP on insomnia (IVW: OR = 1.00, CI: 0.994–1.012, *p* = 0.573; MR-Egger: OR = 0.989, CI: 0.971–1.007, *p* = 0.235; Weighted median: OR = 1.00, CI: 0.986–1.014, *p* = 0.996; RAPS: OR = 1.00, CI: 0.993–1.012, *p* = 0.663) (Table 1). The scatter plot shows the distribution of the effect of single LBP SNP on insomnia (Figure 2C). The forest plot and the plot of the leave-one-out analysis are shown in Supplementary Figures S5, S6. The sensitivity analysis excluded horizontal pleiotropy and heterogeneity (*p* > 0.05, Supplementary Table S3).

### The causal effect of low back pain on daytime sleepiness

In the analysis, 26 SNPs were taken as IVs for LBP. The results of IVW model suggested that LBP was not associated with the risk of daytime sleepiness (OR = 1.00, CI: 0.998–1.010, *p* = 0.235). The Weighted median, MR-Egger and RAPS estimations provided supporting evidence (Figure 2D; Table 2). The results did not display obvious evidence of heterogeneity or pleiotropy (*p* > 0.05, Supplementary Table S3). The forest plot and the plot of the leave-one-out analysis are displayed in Supplementary Figures S7, S8.

### Robustness check

Summary statistics of LBP from UK biobank were included for replication purposes. In the analysis, genetically determined insomnia was positive associated with the risk of LBP (IVW: OR = 1.00, CI: 1.003–1.005, *p* = 9.54e-12), while there was no evidence of a reverse causality. No association was found between daytime sleepiness and LBP. No significant heterogeneity and pleiotropy were found in the replication practice. Specific information could be found in Supplementary Material. Obviously, these results were consistent with that in initial practice, indicating our MR analysis results are robust.

## Discussion

Our findings reported coheritability of LBP to both insomnia and daytime sleepiness. By performing bidirectional MR analyses, our results provided genetic evidence that insomnia causally affects LBP and there is no reverse causality. Specifically, individuals having insomnia have an increased risk for LBP, and LBP patients do not have a statistically significant trend to develop insomnia. In addition, our study found no evidence of a bidirectional causal link between daytime sleepiness and LBP.

In the past decade, substantial studies have shown an association between sleep problems and the risk of chronic pain in the low back and neck/shoulders, as well as chronic widespread pain (Gupta et al., 2007; Canivet et al., 2008; Mork et al., 2014; Uhlig et al., 2018; Skarpsno et al., 2021). A previous prospective cohort study by Agmon and collaborators documented that healthy employed adults with insomnia have a higher risk of experiencing back pain than subjects without insomnia (Agmon and Armon, 2014). A recent study by Ho and coworkers showed that the adjusted risk ratio of chronic LBP in insomnia participants was 1.20 (Ho et al., 2022). On the other hand, it has been reported daytime sleepiness positively correlated with the symptom and frequency of back pain (Gustafsson et al., 2018; Uchmanowicz et al., 2019). The previous MR study conducted by Broberg and coworkers demonstrated that genetic liability to insomnia symptoms is significantly causal to reporting pain, including LBP (Broberg et al., 2021). Take advantage of the UK Biobank as well as the latest FinnGen cohort of LBP, the present study provided more robust evidence that insomnia, not daytime sleepiness, leads to a higher risk for developing LBP. Note that the reduction of sleep problems is accompanied by an improvement in LBP (Pakpour et al., 2018; Skarpsno et al., 2020). Particularly, the treatment of insomnia significantly improved the pain symptoms of patients with LBP in a randomized, double-blind placebo-controlled trial (Goforth et al., 2014).

Although sleep disturbances are becoming increasingly recognized as one of the most reported comorbidities in patients with LBP, the causal relationship of LBP on sleep traits is controversial. Tang et al. (2007) found that individuals with LBP are 18 times more likely to suffer from insomnia. The study by Bahouq et al. (2013) revealed that 78% of patients with chronic LBP experienced insomnia, and in 64% of them, insomnia was caused by LBP. A retrospective study on the Korea population found that 43% of LBP patients developed mild to severe insomnia (Kim et al., 2015). Similarly, studies also have shown that at least 50% of patients with LBP have insomnia at the same time (O’Donoghue et al., 2009; van de Water et al., 2011). However, the LBP was not identified as the predictor for insomnia in a prospective cohort study (Agmon and Armon, 2014). Our findings were in line with this research and did not provide significant genetic evidence for LBP as a risk factor for insomnia. Besides, the causal link of genetically predicted daytime sleepiness on the odds of reporting LBP has not been found.

There are several potential biological mechanisms underlying the link between sleep deficiency and LBP. Sleep deprivation may lead to upregulation of inflammatory mediators, possibly by affecting the immune system, ultimately leading to hyperalgesia (Mullington et al., 2010; da Costa Souza and Ribeiro, 2015). In animal studies, sleep loss increases pain sensation in healthy mice, which is involved in decreasing dopaminergic activity in the nucleus accumbens or increasing adenosinergic activity in median preoptic nucleus (Alexandre et al., 2017; Sardi et al., 2018). Moreover, individuals with insomnia symptoms have a mild increase in basal cortisol levels and a hyper-reactivity of the Hypothalamus-pituitary-adrenal axis, which are associated with pain sensitivity (Balbo et al., 2010; Goodin et al., 2012). On the other hand, various other systems, such as the opioid system, the monoaminergic system and adenosine signaling, mediate the effect of deficient sleep on pain (Haack et al., 2020). Further studies are necessary to investigate the mechanisms underlying the association between insomnia and LBP.

Our study has several strengths. First, this is the first analysis focused on the bidirectional causal relationship between two sleep traits (insomnia, daytime sleepiness) and LBP by MR methods, extending the relevant studies. Second, potential confounders and other biases were removed by genetic variants. Third, potential horizontal pleiotropy was detected and corrected by the MR-PRESSO method.

Some inevitable limitations should also be mentioned. Firstly, our study cannot evaluate the influence of within-population structures resulting from the between-sex difference in the prevalence of insomnia (Aili et al., 2015; Van Looveren et al., 2021). Secondly, MR does not inherently completely expel unknown pleiotropy that affected our results. Finally, the association between sleep disturbances and LBP needs to be further explored in other populations, as this study was restricted to subjects of European ancestries.

In summary, the results of this study reveal a deleterious effect of genetically predicted insomnia on LBP and a null causal effect between daytime sleepiness and LBP. Nonetheless, large-scale longitudinal studies, as well as in-depth mechanistic studies, need to be done. Importantly, the MR result implies that attention should be paid in clinical practice to comorbid insomnia and chronic LBP. A better understanding of the relationship between these two conditions will contribute to pain prevention and improvement in global public health.

## Data Availability

The original contributions presented in the study are included in the article/Supplementary Material, further inquiries can be directed to the corresponding author.
