# Supplementary material for: Association of insomnia and daytime sleepiness with low back pain: A bidirectional mendelian randomization analysis
Source: Front Genet. 2022 Oct 4;13:938334. doi: 10.3389/fgene.2022.938334 (PMC9577110; doi:10.3389/fgene.2022.938334)

Supplementary Figures

Supplementary Figure S1: Forest plot of insomnia with risk of LBP.

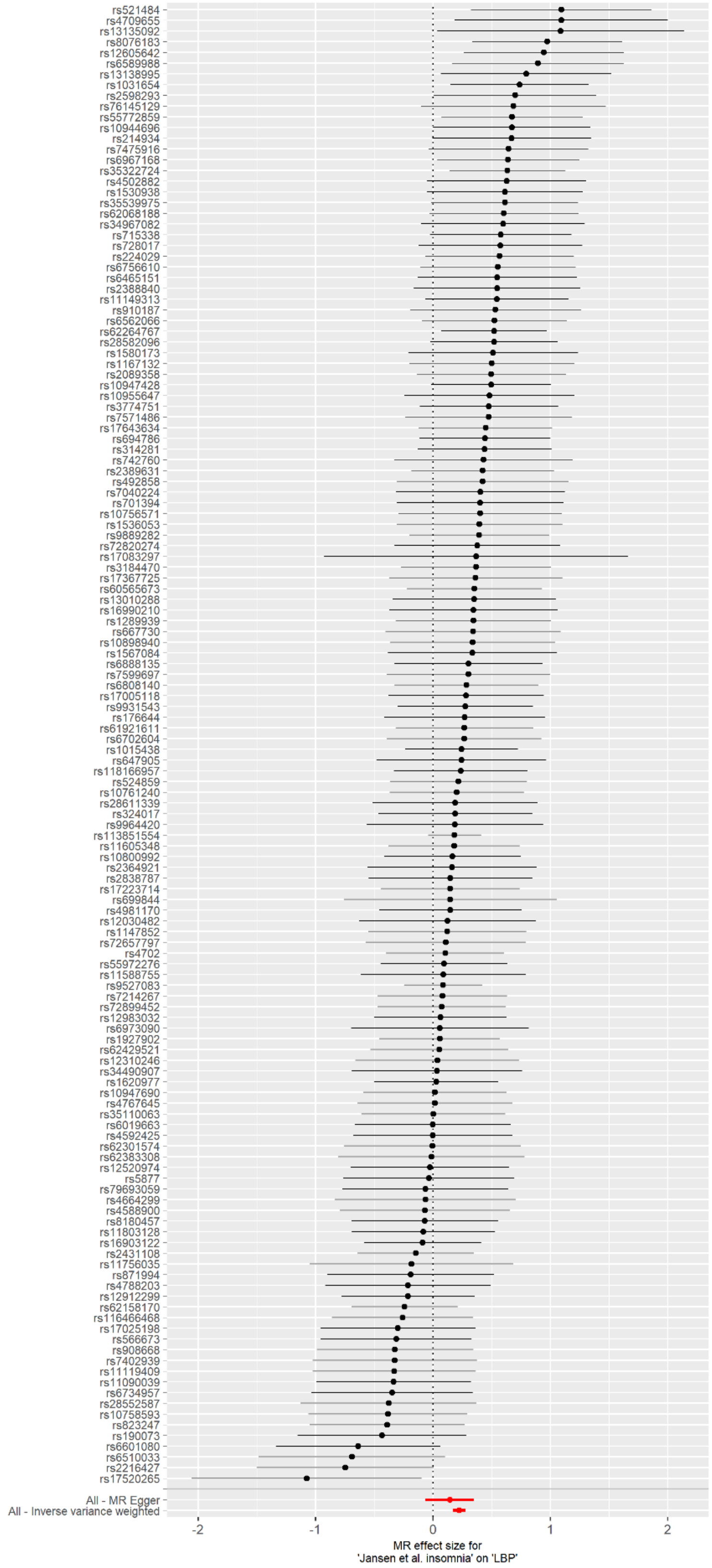

Supplementary Figure S2: Leave-one-out analysis plot of insomnia with risk of LBP.

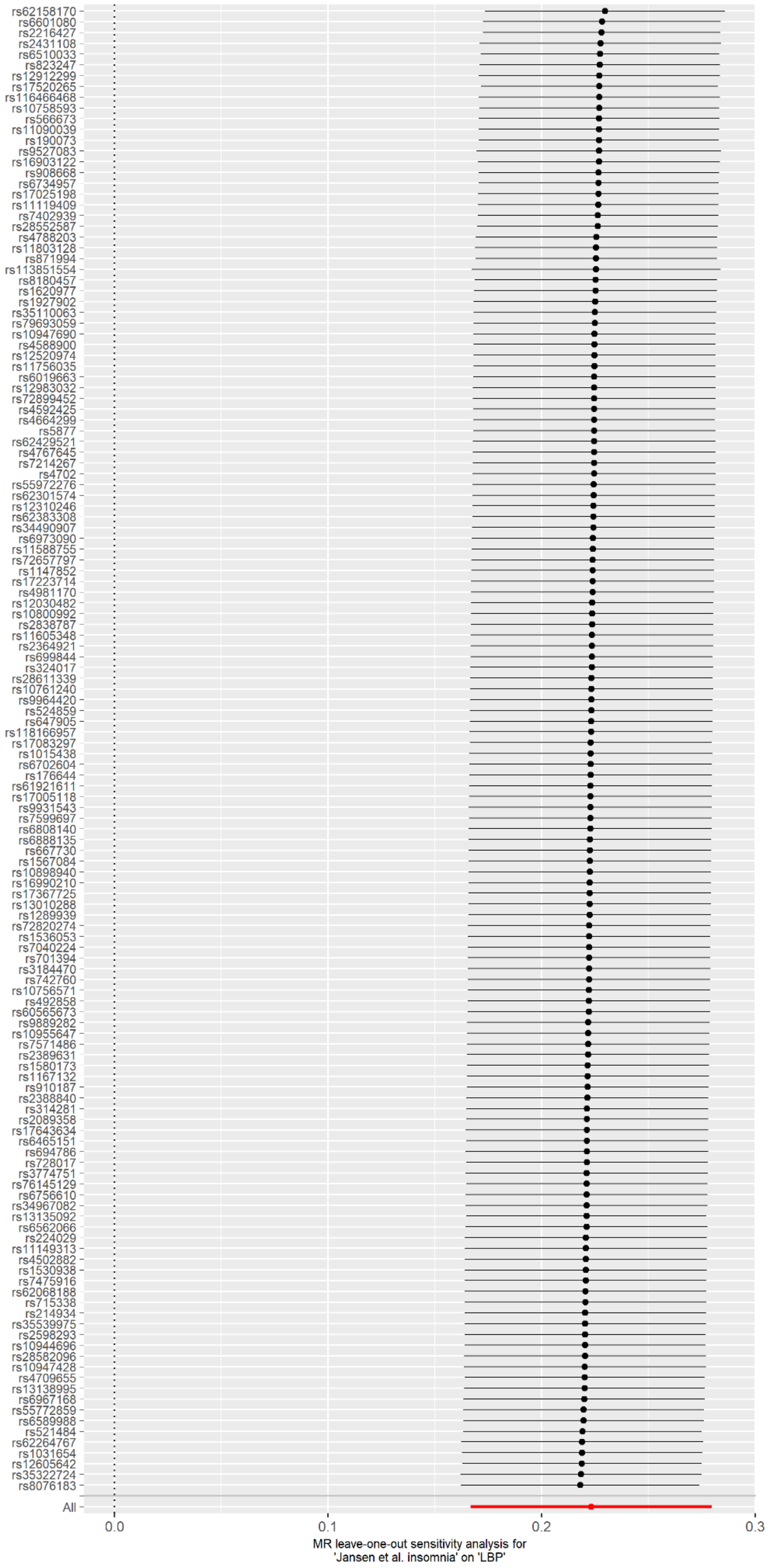

Supplementary Figure S3: Forest plot for daytime sleepiness on low back pain.

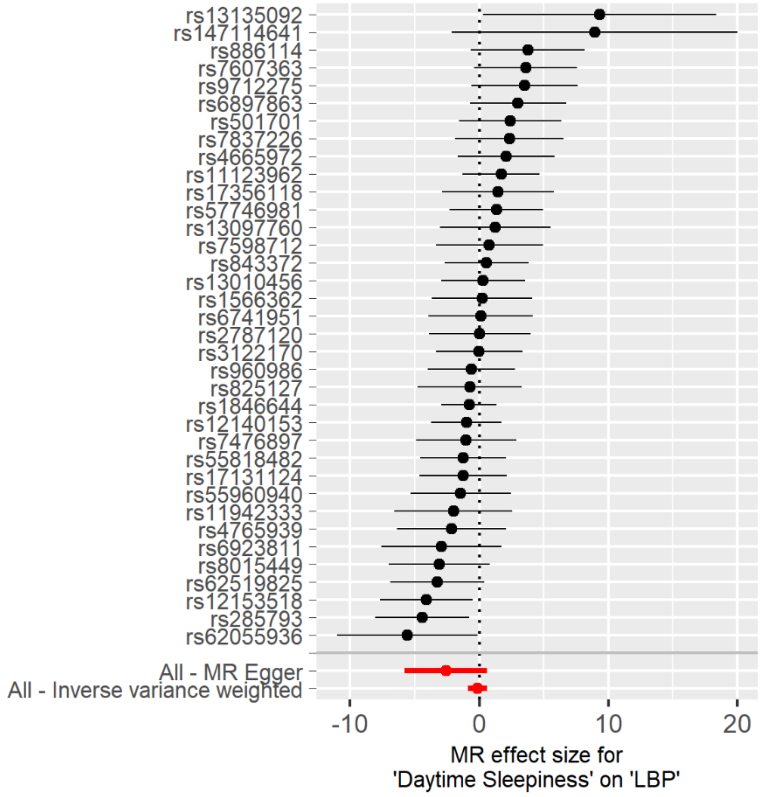

Supplementary Figure S4: Leave-one-out analysis plot for daytime sleepiness on low back pain.

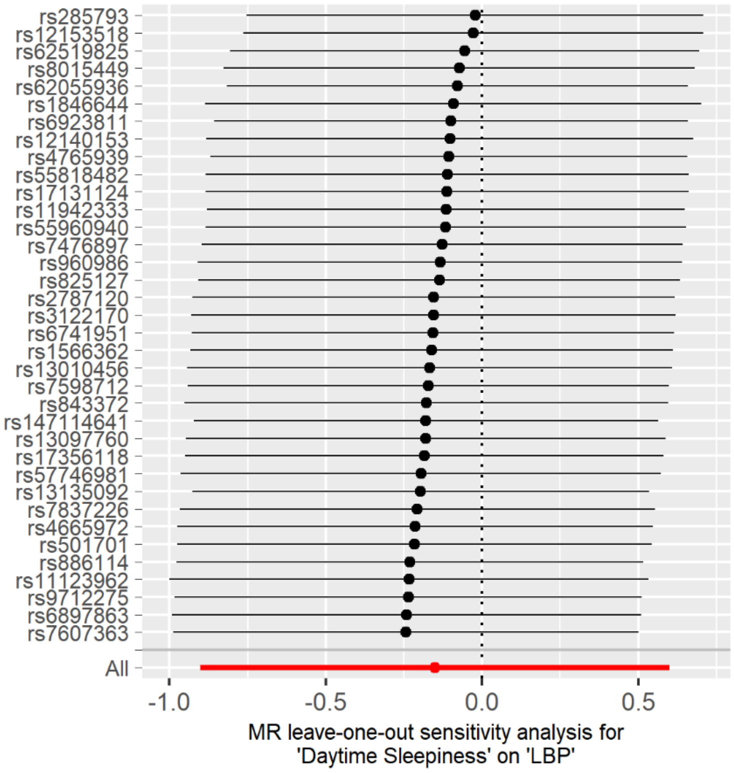

Supplementary Figure S5: Forest plot for low back pain on insomnia

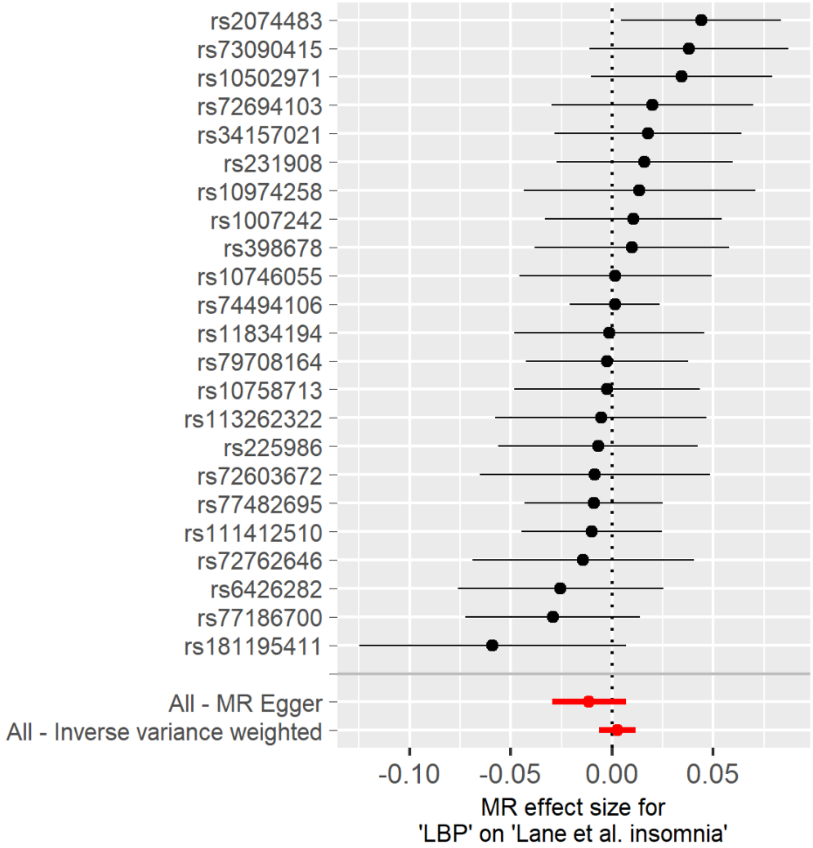

Supplementary Figure S6: Leave-one-out analysis plot for low back pain on insomnia

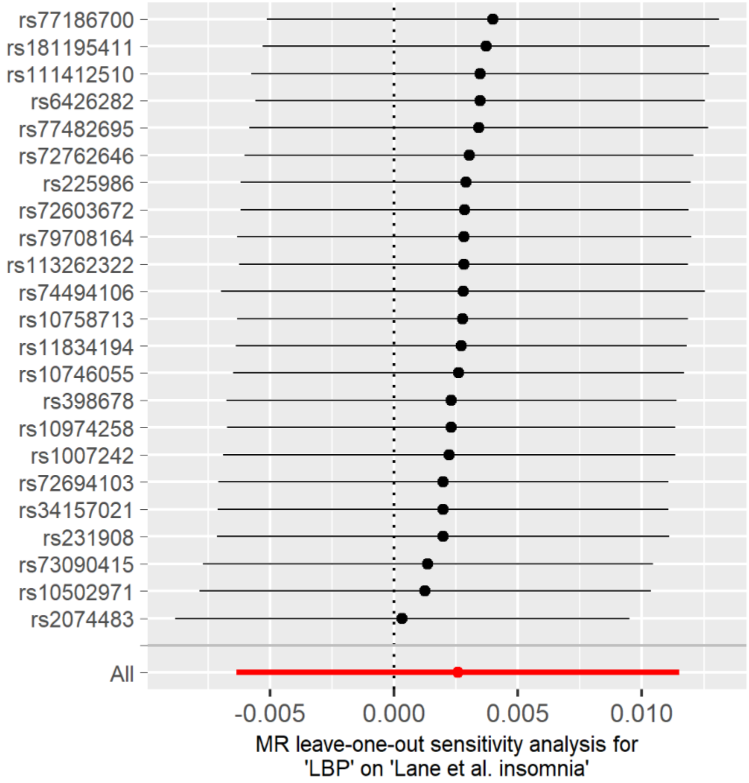

Supplementary Figure S7: Forest plot for low back pain on daytime sleepiness.

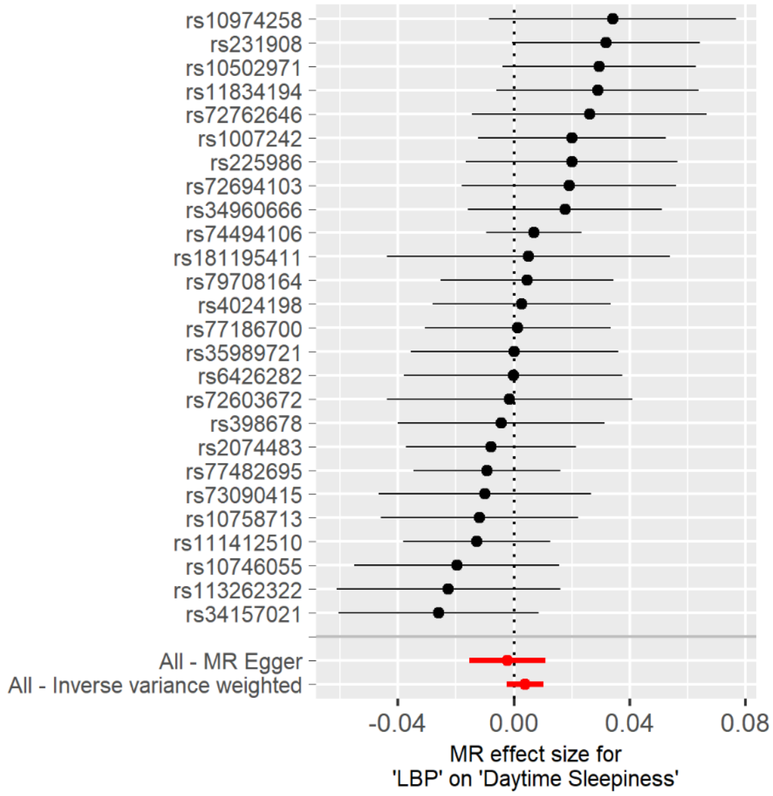

Supplementary Figure S8: Leave-one-out analysis plot for low back pain on daytime sleepiness.

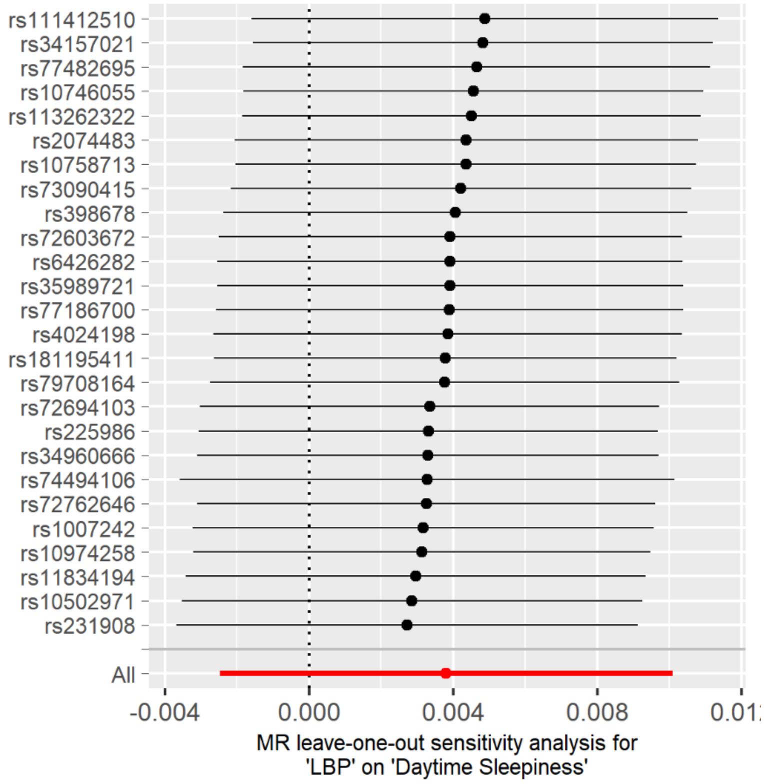

Supplement: Supplementary file 1 [file DataSheet1.zip › Supplementary Figures.pdf]
