# Supplementary material for: Association of insomnia and daytime sleepiness with low back pain: A bidirectional mendelian randomization analysis
Source: Front Genet. 2022 Oct 4;13:938334. doi: 10.3389/fgene.2022.938334 (PMC9577110; doi:10.3389/fgene.2022.938334)
Supplement: Supplementary file 1 [file DataSheet1.zip › Supplementary Tables.pdf]

Supplementary Tables

Supplementary Table S1: Detailed information on included traits in the present study.

Traits	Role	Data source	Sample size	PMID or web source
Insomnia	exposure	Meta-analysis	1,331,010 individuals (386 ,533 from UK Biobank and 944,477 from 23andMe)	PMID: 30804565
	outcome	UKB	453,379 individuals	https://ctg.cncr.nl/software/summary_statistics
Daytime sleepiness		UKB	452,071 individuals	PMID: 31409809
LBP	exposure/outcome	FinnGen R6	16,356 cases and 194,289 controls	https://www.finngen.fi/en
		UKB	5,423 cases and 355,771 controls	https://gwas.mrcieu.ac.uk/datasets

LBP, Low back pain; PMID, PubMed identifier; UKB, UK Biobank.

Supplementary Table S2: All IVs for exposure in the study.

SNP	EA	OA	EAF	Beta	SE	Pval	F
**Insomnia (158 SNPs)**							
rs113851554	T	G	0.051	0.206	0.014	1.56E-51	216.933
rs1064939	A	T	0.978	0.130	0.020	2.16E-10	42.348
rs72899452	T	C	0.065	0.074	0.012	1.00E-09	38.212
rs55972276	A	C	0.137	0.073	0.009	4.19E-17	66.242
rs77641763	T	C	0.122	0.071	0.009	6.53E-15	62.920
rs2286729	A	G	0.086	0.070	0.011	5.37E-11	39.949
rs118166957	T	C	0.159	0.068	0.008	1.95E-16	71.526
rs62158170	A	G	0.786	0.066	0.007	1.20E-19	88.327
rs62264767	A	C	0.853	0.065	0.008	1.63E-14	65.713
rs699844	A	G	0.920	0.060	0.011	4.11E-08	29.905
rs28611339	T	G	0.128	0.058	0.009	8.46E-11	41.917
rs1015438	A	G	0.188	0.058	0.008	2.51E-14	53.051
rs6465151	T	C	0.113	0.056	0.009	1.90E-09	39.244
rs16903122	T	C	0.249	0.055	0.007	9.04E-16	62.714
rs34490907	C	G	0.888	0.054	0.009	1.76E-08	35.390
rs1927902	T	C	0.254	0.053	0.007	1.15E-14	56.448
rs1620977	A	G	0.270	0.052	0.007	2.27E-14	54.429
rs11756035	C	G	0.128	0.051	0.009	1.29E-08	31.726
rs62429521	A	C	0.146	0.051	0.008	1.78E-09	40.153
rs908668	T	C	0.208	0.050	0.007	1.41E-11	50.495

rs35322724	A	C	0.577	0.049	0.006	3.75E-16	66.124
rs62068188	T	C	0.834	0.049	0.008	1.18E-09	37.195
rs9931543	T	C	0.736	0.048	0.007	1.11E-12	46.702
rs17223714	A	G	0.789	0.046	0.007	2.44E-10	43.050
rs429358	T	C	0.846	0.046	0.008	2.13E-08	32.960
rs12310246	A	G	0.249	0.045	0.007	4.74E-11	41.278
rs830716	C	G	0.713	0.045	0.007	8.68E-12	41.278
rs116466468	T	C	0.759	0.044	0.007	2.11E-10	39.541
rs742760	A	T	0.816	0.043	0.008	2.48E-08	28.971
rs10800992	T	C	0.443	0.042	0.006	3.84E-12	49.236
rs55772859	A	C	0.311	0.042	0.006	4.82E-11	49.236
rs17005118	A	G	0.264	0.042	0.007	6.13E-10	36.174
rs35539975	A	G	0.779	0.042	0.007	4.49E-09	36.174
rs12666306	A	G	0.502	0.042	0.006	2.24E-12	49.236
rs12030482	A	T	0.220	0.041	0.007	8.16E-09	34.544
rs17025198	A	G	0.204	0.041	0.007	2.19E-08	34.544
rs715338	A	G	0.578	0.041	0.006	7.85E-12	47.018
rs12991815	C	G	0.424	0.040	0.006	3.02E-11	44.849
rs4592425	T	G	0.697	0.040	0.006	4.31E-10	44.849
rs11149313	A	G	0.730	0.040	0.007	2.38E-09	32.951
rs6019663	T	C	0.293	0.040	0.007	6.47E-10	32.951
rs6808140	T	C	0.505	0.039	0.006	5.35E-11	42.730
rs35110063	A	G	0.427	0.039	0.006	8.82E-11	42.730
rs1147852	A	G	0.310	0.039	0.006	9.94E-10	42.730
rs324017	A	C	0.294	0.039	0.007	1.61E-09	31.393
rs6562066	T	C	0.369	0.039	0.006	1.38E-10	42.730
rs1038093	T	C	0.628	0.039	0.006	2.47E-10	42.730
rs11090039	A	G	0.287	0.039	0.007	1.82E-09	31.393
rs6888135	A	C	0.497	0.038	0.006	1.21E-10	40.659
rs6756610	C	G	0.629	0.037	0.006	1.14E-09	38.638
rs7040224	A	G	0.316	0.037	0.006	4.24E-09	38.638
rs5877	T	C	0.669	0.036	0.006	1.23E-08	36.667
rs1530938	A	G	0.442	0.036	0.006	8.82E-10	36.667
rs10756571	T	C	0.685	0.036	0.006	1.80E-08	36.667
rs2221119	C	G	0.443	0.036	0.006	2.00E-09	36.667
rs34967082	A	G	0.414	0.035	0.006	4.34E-09	34.745
rs2216427	C	G	0.653	0.035	0.006	1.60E-08	34.745
rs6601080	A	G	0.676	0.035	0.006	2.21E-08	34.745
rs2598293	T	C	0.476	0.035	0.006	2.48E-09	34.745
rs871994	A	C	0.435	0.035	0.006	5.50E-09	34.745
rs1167132	T	C	0.392	0.035	0.006	8.73E-09	34.745
rs176644	T	G	0.404	0.035	0.006	9.49E-09	34.745
rs12605642	T	G	0.486	0.035	0.006	2.13E-09	34.745
rs9964420	A	C	0.301	0.035	0.007	4.54E-08	25.527

rs72820274	A	G	0.417	0.034	0.006	1.28E-08	32.874
rs13138995	A	G	0.390	0.034	0.006	1.97E-08	32.874
rs2030672	C	G	0.559	0.034	0.006	1.10E-08	32.874
rs10898940	A	C	0.517	0.034	0.006	8.09E-09	32.874
rs1567084	A	G	0.498	0.033	0.006	2.14E-08	31.052
rs1580173	A	G	0.561	0.033	0.006	2.28E-08	31.052
rs4588900	A	G	0.516	0.033	0.006	1.57E-08	31.052
rs28552587	A	G	0.564	0.033	0.006	3.30E-08	31.052
rs10955647	T	G	0.532	0.033	0.006	1.84E-08	31.052
rs667730	T	C	0.579	0.033	0.006	2.26E-08	31.052
rs647905	T	C	0.541	0.033	0.006	2.87E-08	31.052
rs4858708	A	T	0.531	-0.034	0.006	1.23E-08	31.279
rs2364921	T	C	0.469	-0.034	0.006	2.13E-08	31.279
rs190073	A	G	0.414	-0.034	0.006	2.86E-08	31.279
rs11588755	A	G	0.522	-0.035	0.006	5.14E-09	33.238
rs11119409	T	C	0.587	-0.035	0.006	1.19E-08	33.238
rs728017	A	G	0.386	-0.035	0.006	9.51E-09	33.238
rs1731951	A	T	0.443	-0.035	0.006	1.36E-08	33.238
rs4788203	A	G	0.433	-0.035	0.006	6.32E-09	33.238
rs910187	A	G	0.373	-0.035	0.006	1.63E-08	33.238
rs12520974	T	C	0.485	-0.036	0.006	1.69E-09	35.258
rs701394	A	G	0.638	-0.036	0.006	6.83E-09	35.258
rs17367725	T	C	0.351	-0.036	0.006	9.29E-09	35.258
rs10758593	A	G	0.399	-0.036	0.006	4.90E-09	35.258
rs7402939	T	C	0.376	-0.036	0.006	5.19E-09	35.258
rs2838787	A	G	0.392	-0.036	0.006	7.65E-09	35.258
rs6702604	A	G	0.584	-0.037	0.006	1.30E-09	37.340
rs823247	T	C	0.479	-0.037	0.006	5.25E-10	37.340
rs7599697	T	C	0.358	-0.037	0.006	5.00E-09	37.340
rs2388840	A	G	0.576	-0.037	0.006	1.37E-09	37.340
rs7475916	C	G	0.353	-0.037	0.006	6.70E-09	37.340
rs4767645	T	G	0.461	-0.037	0.006	6.47E-10	37.340
rs6510033	A	G	0.725	-0.037	0.007	4.66E-08	27.434
rs10944696	A	G	0.298	-0.038	0.007	7.99E-09	29.009
rs6973090	A	G	0.250	-0.038	0.007	4.31E-08	29.009
rs671985	A	G	0.452	-0.038	0.006	2.79E-10	39.484
rs11001276	A	T	0.740	-0.038	0.007	2.52E-08	29.009
rs214934	A	T	0.312	-0.038	0.006	3.16E-09	39.484
rs6589988	A	G	0.676	-0.038	0.006	4.70E-09	39.484
rs1536053	T	C	0.316	-0.038	0.006	6.04E-09	39.484
rs3184470	A	G	0.351	-0.038	0.006	9.73E-10	39.484
rs8076183	T	C	0.449	-0.038	0.006	2.75E-10	39.484
rs7571486	A	G	0.251	-0.039	0.007	1.40E-08	30.630
rs4502882	T	C	0.658	-0.039	0.006	7.96E-10	41.690

rs12251016	A	T	0.656	-0.039	0.006	3.89E-10	41.690
rs224029	T	C	0.400	-0.039	0.006	2.51E-10	41.690
rs566673	T	G	0.535	-0.039	0.006	1.18E-10	41.690
rs521484	A	G	0.767	-0.040	0.007	1.53E-08	32.296
rs2389631	A	C	0.667	-0.040	0.006	2.03E-10	43.959
rs2089358	T	C	0.704	-0.041	0.007	2.75E-10	34.009
rs1289939	T	C	0.233	-0.041	0.007	6.00E-09	34.009
rs11803128	A	G	0.654	-0.041	0.006	6.85E-11	46.290
rs4664299	T	C	0.235	-0.041	0.007	4.95E-09	34.009
rs3774751	T	G	0.462	-0.041	0.006	7.32E-12	46.290
rs7044885	C	G	0.442	-0.041	0.006	5.67E-12	46.290
rs6734957	T	G	0.239	-0.042	0.007	1.82E-09	35.768
rs62301574	C	G	0.800	-0.042	0.007	1.37E-08	35.768
rs9889282	A	C	0.613	-0.042	0.006	4.70E-12	48.684
rs314281	T	C	0.453	-0.043	0.006	6.03E-13	51.140
rs10761240	A	G	0.396	-0.043	0.006	2.12E-12	51.140
rs12912299	T	C	0.489	-0.043	0.006	4.42E-13	51.140
rs60565673	T	G	0.621	-0.043	0.006	1.59E-12	51.140
rs12983032	A	G	0.343	-0.043	0.006	1.07E-11	51.140
rs694786	T	C	0.461	-0.044	0.006	1.97E-13	53.660
rs17083297	A	C	0.177	-0.044	0.008	1.60E-08	30.184
rs6967168	T	G	0.754	-0.044	0.007	1.39E-10	39.424
rs524859	A	G	0.360	-0.044	0.006	1.48E-12	53.660
rs61921611	T	C	0.692	-0.044	0.006	7.84E-12	53.660
rs7214267	A	G	0.581	-0.044	0.006	5.09E-13	53.660
rs11605348	A	G	0.350	-0.045	0.006	7.01E-13	56.243
rs16990210	T	C	0.848	-0.046	0.008	1.97E-08	33.126
rs10947690	A	G	0.741	-0.047	0.007	4.04E-12	45.258
rs4702	A	G	0.556	-0.048	0.006	6.78E-16	64.375
rs8180817	C	G	0.430	-0.049	0.006	1.83E-16	67.213
rs76145129	T	G	0.124	-0.050	0.009	2.73E-08	31.163
rs1031654	A	C	0.800	-0.051	0.007	3.88E-12	53.694
rs2431108	T	C	0.672	-0.053	0.006	7.83E-17	79.212
rs4709655	T	C	0.119	-0.054	0.009	3.09E-09	36.611
rs28582096	A	G	0.205	-0.054	0.007	1.74E-13	60.520
rs4981170	A	G	0.194	-0.054	0.008	7.33E-13	46.336
rs72657797	T	C	0.176	-0.056	0.008	1.52E-12	48.151
rs8180457	T	C	0.157	-0.056	0.008	1.12E-11	48.151
rs73671843	A	G	0.126	-0.056	0.009	5.49E-10	38.045
rs13010288	T	G	0.133	-0.060	0.009	9.26E-12	44.075
rs62383308	A	G	0.081	-0.060	0.011	3.98E-08	29.505
rs17643634	T	C	0.165	-0.060	0.008	1.34E-13	55.782
rs66674044	A	T	0.857	-0.060	0.009	2.18E-12	44.075
rs6119267	C	G	0.689	-0.060	0.006	2.32E-20	99.168

rs492858	T	C	0.076	-0.066	0.011	3.46E-09	36.153
rs10947428	T	C	0.786	-0.068	0.007	9.06E-21	95.143
rs79693059	C	G	0.916	-0.073	0.011	1.61E-11	43.525
rs9527083	A	G	0.671	-0.076	0.006	1.61E-32	159.608
rs13135092	A	G	0.918	-0.089	0.011	2.53E-16	65.215
rs17520265	A	G	0.034	-0.091	0.016	2.87E-08	32.361
**Daytime sleepiness (38 SNPs)**							
rs2787120	A	G	0.833	0.008	0.001	2.00E-08	31.929
rs12140153	G	T	0.905	0.017	0.002	2.80E-20	85.186
rs501701	A	G	0.531	0.006	0.001	6.50E-09	34.161
rs17131124	C	G	0.912	-0.011	0.002	1.70E-09	37.027
rs57746981	C	T	0.644	0.007	0.001	2.20E-10	40.068
rs825127	T	G	0.531	0.006	0.001	9.50E-09	32.781
rs4665972	T	C	0.394	0.007	0.001	3.90E-10	39.165
rs7598712	G	T	0.555	0.006	0.001	2.20E-08	30.923
rs6741951	G	A	0.711	0.007	0.001	2.70E-09	35.683
rs11123962	T	G	0.553	-0.008	0.001	7.50E-15	60.480
rs9712275	C	T	0.486	-0.006	0.001	1.30E-08	32.515
rs7607363	A	G	0.561	-0.006	0.001	8.00E-09	33.658
rs13010456	A	G	0.595	0.008	0.001	2.10E-13	54.229
rs13097760	A	C	0.639	-0.006	0.001	3.20E-08	30.864
rs960986	C	T	0.637	0.007	0.001	1.50E-11	44.950
rs843372	C	T	0.230	0.008	0.001	2.20E-11	44.342
rs11942333	G	A	0.676	-0.006	0.001	3.80E-08	30.106
rs13135092	A	G	0.917	-0.010	0.002	3.10E-08	30.277
rs6897863	A	C	0.584	0.006	0.001	7.60E-10	38.012
rs12153518	A	G	0.472	0.007	0.001	6.80E-11	42.213
rs6923811	T	C	0.679	0.007	0.001	9.10E-10	37.569
rs55960940	T	C	0.822	0.008	0.001	2.00E-08	31.838
rs3122170	C	A	0.231	0.010	0.001	5.60E-15	60.001
rs62519825	T	C	0.887	-0.009	0.002	3.80E-09	33.892
rs285793	G	A	0.461	0.007	0.001	7.90E-11	42.685
rs7837226	A	G	0.473	-0.006	0.001	2.00E-08	30.916
rs55818482	T	C	0.785	-0.010	0.001	1.40E-14	59.702
rs1566362	T	C	0.632	0.006	0.001	3.80E-09	34.971
rs7476897	G	A	0.679	0.007	0.001	2.70E-11	45.466
rs4765939	G	C	0.583	-0.006	0.001	2.00E-09	36.012
rs1846644	T	C	0.591	-0.011	0.001	2.50E-27	117.439
rs8015449	A	G	0.539	0.006	0.001	1.90E-09	35.813
rs17356118	A	G	0.769	-0.008	0.001	2.60E-10	39.320
rs886114	C	T	0.357	0.006	0.001	1.90E-08	31.640
rs11078398	G	A	0.744	0.008	0.001	7.10E-10	38.089
rs147114641	C	A	0.774	0.008	0.001	1.70E-10	41.038
rs62055936	T	A	0.777	0.008	0.001	4.40E-11	43.648

rs2048522	A	T	0.565	0.006	0.001	3.50E-08	30.667
**LBP (FinnGen R6 ,26 SNPs)**							
rs6426282	C	T	0.195	-0.078	0.015	4.81E-07	25.351
rs74494106	T	C	0.005	-0.440	0.091	1.45E-06	23.213
rs34157021	C	T	0.094	0.101	0.021	1.79E-06	22.813
rs72694103	A	G	0.123	-0.089	0.019	2.52E-06	22.149
rs2074483	A	G	0.300	0.070	0.013	1.98E-07	27.043
rs35989721	C	T	0.102	0.133	0.020	4.66E-11	43.304
rs113262322	T	C	0.045	0.148	0.031	1.22E-06	23.547
rs73090415	C	T	0.242	0.067	0.014	2.76E-06	21.969
rs111412510	T	C	0.005	0.463	0.090	2.87E-07	26.334
rs77482695	G	A	0.018	0.224	0.047	2.33E-06	22.305
rs10974258	C	T	0.019	0.213	0.045	2.76E-06	21.978
rs10758713	G	A	0.909	0.102	0.021	1.97E-06	22.618
rs72603672	A	T	0.158	0.080	0.017	1.62E-06	22.991
rs72762646	C	T	0.161	-0.082	0.017	1.08E-06	23.793
rs4024198	G	C	0.715	-0.072	0.014	1.02E-07	28.337
rs1007242	G	A	0.445	0.062	0.012	4.21E-07	25.595
rs231908	G	A	0.754	-0.075	0.014	1.83E-07	27.195
rs11834194	T	G	0.157	0.079	0.017	3.46E-06	21.556
rs34960666	A	G	0.411	-0.061	0.012	1.06E-06	23.797
**LBP (UK Biobank, 20 SNPs)**							
rs56208139	A	T	0.052	-0.003	0.001	4.59E-06	21.001
rs12135248	G	A	0.034	0.004	0.001	2.83E-06	21.931
rs115939263	T	G	0.007	0.009	0.002	7.56E-07	24.466
rs113592230	G	T	0.154	0.002	0.000	3.80E-06	21.366
rs111995428	A	G	0.019	0.005	0.001	4.43E-06	21.072
rs4413294	C	T	0.162	-0.002	0.000	3.22E-06	21.684
rs551380723	A	G	0.011	0.007	0.001	4.75E-07	25.364
rs150035458	C	T	0.013	0.006	0.001	3.35E-06	21.604
rs6871675	C	T	0.809	0.002	0.000	4.83E-06	20.904
rs112298683	T	C	0.011	0.007	0.001	4.24E-06	21.152
rs140484435	C	G	0.087	0.003	0.001	8.63E-07	24.212
rs73145372	T	C	0.029	0.004	0.001	2.78E-06	21.962
rs145573211	C	T	0.006	0.010	0.002	1.80E-06	22.802
rs10090865	G	A	0.786	0.002	0.000	4.65E-06	20.976
rs13263458	T	C	0.260	0.001	0.000	4.70E-06	20.958
rs17248764	G	A	0.089	-0.002	0.001	2.30E-06	22.324
rs192107903	T	C	0.007	0.008	0.002	2.18E-06	22.430
rs76341560	A	G	0.032	0.004	0.001	2.91E-06	21.875
rs1489166	T	C	0.012	0.006	0.001	4.60E-06	20.998
rs4807174	A	G	0.586	0.001	0.000	1.08E-06	23.772

LBP, low back pain; SNP, single nucleic acid polymorphism; EA, the effect allele; OA, the other allele; EAF, the effect allele frequency; Beta, the effect size; SE, the standard error of the effect size; Pval, The P-value for the SNP's association with the exposure. F, F-statistics = (Beta/SE) ^2.

Supplementary Table S3: Pleiotropy and Heterogeneity test.

	MR Egger regression			Cochran's Q test					
				MR Egger			IVW		
	intercept	SE	pval	Q	Q_df	pval	Q	Q_df	pval
Insomnia on LBP	0.004	0.005	0.435	154.072	136	0.138	154.767	137	0.142
DS on LBP	0.018	0.012	0.135	47.382	34	0.063	50.656	35	0.042
LBP on insomnia	0.001	0.001	0.099	15.241	21	0.811	18.213	22	0.693
LBP on DS	0.001	0.001	0.317	23.647	24	0.482	24.691	25	0.480

LBP, low back pain; DS, daytime sleepiness; SE, standard error.
